# *Mycobacterium tuberculosis* RD-Rio Strain in Kazakhstan

**DOI:** 10.3201/eid2503.181179

**Published:** 2019-03

**Authors:** Yuriy Skiba, Igor Mokrousov, Dilyara Nabirova, Anna Vyazovaya, Elina Maltseva, Natalya Malakhova, Gulnara Ismagulova, Ilva Pole, Renate Ranka, Zhanar Sapiyeva, Shakhimurat Ismailov, Daphne Moffett

**Affiliations:** Almaty Branch of National Center for Biotechnology at Central Reference Laboratory, Almaty, Kazakhstan (Y. Skiba);; Aitkhozhin Institute of Molecular Biology and Biochemistry, Almaty (Y. Skiba, E. Maltseva, N. Malakhova, G. Ismagulova);; St. Petersburg Pasteur Institute, St. Petersburg, Russia (I. Mokrousov, A. Vyazovaya);; Centers for Disease Control and Prevention, Central Asia Regional Office, Almaty (D. Nabirova, D. Moffett);; Latvian Biomedical Research and Study Centre, Riga, Latvia (I. Pole, R. Ranka);; Ministry of Health, Almaty (Z. Sapiyeva, S. Ismailov);; The Global Fund, Geneva, Switzerland (S. Ismailov)

**Keywords:** tuberculosis and other mycobacteria, molecular epidemiology, mycobacteria, bacteria, drug resistance, antimicrobial resistance, RD-Rio, Kazakhstan

## Abstract

*Mycobacterium tuberculosis* RD-Rio strains are still rare in the former Soviet Union countries and Asia. We describe a strain in Kazakhstan that belongs to the RD-Rio secondary branch, which is endemic to northwest Russia and eastern Europe. Although RD-Rio strains are frequently multidrug resistant, this heterogeneous branch included only drug-susceptible isolates.

The RD-Rio strain of *Mycobacterium tuberculosis* was initially described in Rio de Janeiro, Brazil, and was demonstrated to be spread beyond South America (*1*). However, RD-Rio isolates are rare in northern Eurasia (i.e., Baltic and former Soviet Union countries) (*2*). Phylogenetically, RD-Rio is part of the Latin-American-Mediterranean (LAM) genetic family and is marked by 2 large genomic deletions, RD174 and RD-Rio (*3*); RD-Rio is speculatively associated with particular pathogenic properties (*1*).

We describe a strain from Kazakhstan with confirmed RD-Rio deletion. Molecular analysis and comparison with the global LAM dataset showed that it belongs to the particular secondary branch described in the north of European Russia and Eastern Europe. Although RD-Rio isolates have been associated with multidrug resistance (MDR) (*4*), this branch on a dendrogram (Figure, panel A) included only drug-susceptible isolates.

**Figure:**
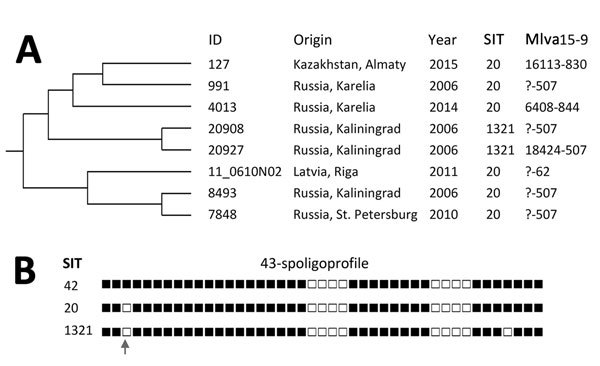
*Mycobacterium tuberculosis* RD-Rio strain in Kazakhstan. A) Section of the variable-number tandem-repeat–based dendrogram of the Latin-American-Mediterranean family of *Mycobacterium tuberculosis* RD-Rio strain with enlarged branch including SIT20 strain from Kazakhstan. All isolates were drug susceptible. The complete dendrogram with VNTR profiles is provided in Appendix Figures 1,2. B) Binary spoligoprofiles of the studied strains.

We conducted this study as part of an ongoing molecular epidemiologic surveillance study of *M. tuberculosis* in Kazakhstan implemented in collaboration with the Centers for Disease Control and Prevention office in Kazakhstan (CDC-Central Asia Region Office). *M. tuberculosis* strain #127 was isolated in Almaty, Kazakhstan, in 2015 from a 52-year-old man with pulmonary tuberculosis (TB). He received anti-TB treatment at the TB hospital of the Interdistrict TB Dispensary in Almaty for 2 months and was discharged. We tested the strain for drug susceptibility to the first- and second-line drugs (streptomycin, isoniazid, rifampin, ethambutol, prothionamide, ofloxacin, kanamycin, capreomycin, and cycloserine). We tested DNA for drug-resistance mutations (in *rpoB*, *katG*, *inhA*, *ahpC*, *embB*, *gyrA*, *gyrB*, *rrs*, *eis*); spoligotyping; 24-locus variable-number tandem-repeat (VNTR) typing; detection of genome deletions RD174, RD-Rio, and RD115; and LAM family–specific single-nucleotide polymorphisms (SNPs) in *Rv0129c* (Appendix). The strain was susceptible to all tested drugs and did not bear drug resistance mutations in the tested gene targets. It was assigned to the RD-Rio sublineage, spoligotype SIT20 (according to SITVIT2 database, http://www.pasteur-guadeloupe.fr:8081/SITVIT2/), and Mlva15-9 code #16113-830 (according to https://www.MIRU-VNTRplus.org). We conducted phylogenetic analysis on the 24-MIRU-VNTR profile of this strain along with 357 isolates of the global LAM dataset (*5*) (Figure; Appendix Figure 1, 2).

A recent global LAM study demonstrated that SIT20 is one of the major RD-Rio spoligotypes and is subdivided into 2 branches on the basis of the ETRB locus alleles (*5*). In this study, we showed that, on the global LAM tree, the strain from Kazakhstan clustered within the branch that included only drug-susceptible isolates from northwestern Russia and Latvia with SIT20 and derived SIT1321 spoligoprofiles (Figure, panel B; Appendix Figure 1).

The case-patient’s medical record contained no information about his contacts and travel before hospitalization. As a man of working age, he could have traveled to Russia as a migrant worker. It has been estimated that ≈1.9 million Kazakhstan citizens lived in Russia during 1989–2007 (http://focus-migration.hwwi.de/Russian-Federation.6337.0.html?&L=1). In 2015, a total of 2,560,000 persons, including seasonal labor migrants (62% of all outgoing migrants), were known to have migrated from Kazakhstan to Russia (https://www.iom.int/world-migration). Russian law requires that migrants with TB be deported, which may explain why this case-patient preferred to disappear or remain unavailable. Thus, a hypothesis about Russian origin of this strain is based solely on the fact that Kazakhstan is the most common country of origin of immigrants to Russia. However, phylogenetic analysis based on high-resolution VNTR loci placed this strain within the branch exclusively made of the isolates from different and neighboring regions in northwestern Russia and Latvia (Figure). We consider this clustering to be evidence that this strain is related to the *M. tuberculosis* population in the European part of Russia. Another example of cross-country *M. tuberculosis* transmission is the “successful Russian strain” Beijing B0/W148-cluster; its overall prevalence in Kazakhstan is low, at 3%, and its isolates were identified in the northern part of the country that is close to Russia (*6*).

Previously, strains of the LAM RD-Rio or SIT20 spoligotype were not described in several countrywide studies in Kazakhstan during 1997–2014 (*2**,**6**–**8*). In neighboring Kyrgyzstan, SIT20 was not described either in the civilian or penitentiary settings (*9**,**10*). In view of the rarity of RD-Rio isolates in northern Eurasia and their previous absence in Kazakhstan, the isolation of such a strain in Kazakhstan, especially in the most distant southern region, deserves attention. That no other isolates have been found through our ongoing surveillance strongly suggests the strain was imported and not acquired in Kazakhstan. The isolates in this SIT20 Russian branch were sufficiently heterogeneous in terms of VNTR locus diversity; they were isolated in different years, and all isolates were drug susceptible (Figure). RD-Rio is known to be associated with MDR, and even by chance, some of these isolates in former Soviet Union countries could have acquired drug resistance under the current adverse conditions of TB control in this region. Nevertheless, these strains have remained drug susceptible. Further surveillance will be needed to determine if additional strains appear and, if so, whether they remain drug susceptible or acquire drug resistance.

Appendix Additional information about *Mycobacterium tuberculosis* RD-Rio strain in Kazakhstan.

## References

[1] Gibson AL, Huard RC, Gey van Pittius NC, Lazzarini LC, Driscoll J, Kurepina N, et al. Application of sensitive and specific molecular methods to uncover global dissemination of the major RDRio Sublineage of the Latin American-Mediterranean *Mycobacterium tuberculosis* spoligotype family. J Clin Microbiol. 2008;46:1259–67. 10.1128/JCM.02231-07 18234868 PMC2292928

[2] Mokrousov I, Vyazovaya A, Narvskaya O. *Mycobacterium tuberculosis* Latin American-Mediterranean family and its sublineages in the light of robust evolutionary markers. J Bacteriol. 2014;196:1833–41. 10.1128/JB.01485-13 24584500 PMC4011003

[3] Lazzarini LC, Huard RC, Boechat NL, Gomes HM, Oelemann MC, Kurepina N, et al. Discovery of a novel *Mycobacterium tuberculosis* lineage that is a major cause of tuberculosis in Rio de Janeiro, Brazil. J Clin Microbiol. 2007;45:3891–902. 10.1128/JCM.01394-07 17898156 PMC2168543

[4] Dalla Costa ER, Lazzarini LC, Perizzolo PF, Díaz CA, Spies FS, Costa LL, et al. *Mycobacterium tuberculosis* of the RDRio genotype is the predominant cause of tuberculosis and associated with multidrug resistance in Porto Alegre City, South Brazil. J Clin Microbiol. 2013;51:1071–7. 10.1128/JCM.01511-12 23325819 PMC3666761

[5] Mokrousov I, Vyazovaya A, Iwamoto T, Skiba Y, Pole I, Zhdanova S, et al. Latin-American-Mediterranean lineage of *Mycobacterium tuberculosis*: Human traces across pathogen’s phylogeography. Mol Phylogenet Evol. 2016;99:133–43. 10.1016/j.ympev.2016.03.020 27001605

[6] Skiba Y, Mokrousov I, Ismagulova G, Maltseva E, Yurkevich N, Bismilda V, et al. Molecular snapshot of *Mycobacterium tuberculosis* population in Kazakhstan: a country-wide study. Tuberculosis (Edinb). 2015;95:538–46. 10.1016/j.tube.2015.04.012 26076582

[7] Kubica T, Agzamova R, Wright A, Aziz MA, Rakishev G, Bismilda V, et al. The Beijing genotype is a major cause of drug-resistant tuberculosis in Kazakhstan. Int J Tuberc Lung Dis. 2005;9:646–53. 15971392

[8] Ibrayeva A, Kozhamkulov U, Raiymbek D, Alenova A, Igilikova S, Zholdybayeva E, et al. Molecular epidemiology of *Mycobacterium tuberculosis* strains circulating in the penitentiary system of Kazakhstan. [short communication]. Int J Tuberc Lung Dis. 2014;18:298–301. 10.5588/ijtld.13.0558 24670565

[9] Mokrousov I, Valcheva V, Sovhozova N, Aldashev A, Rastogi N, Isakova J. Penitentiary population of *Mycobacterium tuberculosis* in Kyrgyzstan: exceptionally high prevalence of the Beijing genotype and its Russia-specific subtype. Infect Genet Evol. 2009;9:1400–5. 10.1016/j.meegid.2009.07.007 19647804

[10] Mokrousov I, Isakova J, Valcheva V, Aldashev A, Rastogi N. Molecular snapshot of *Mycobacterium tuberculosis* population structure and drug-resistance in Kyrgyzstan. Tuberculosis (Edinb). 2013;93:501–7. 10.1016/j.tube.2013.05.008 23890973

