# Supplementary material for: Mycobacterium tuberculosis RD-Rio Strain in Kazakhstan
Source: Emerg Infect Dis. 2019 Mar;25(3):604–6. doi: 10.3201/eid2503.181179 (PMC6390763; doi:10.3201/eid2503.181179)
Supplement: Appendix — Additional information about Mycobacterium tuberculosis RD-Rio strain in Kazakhstan. [file 18-1179-Techapp-s1.pdf]

Article DOI: https://doi.org/10.3201/eid2503.181179

Mycobacterium tuberculosis RD-Rio Strain in Kazakhstan

Appendix

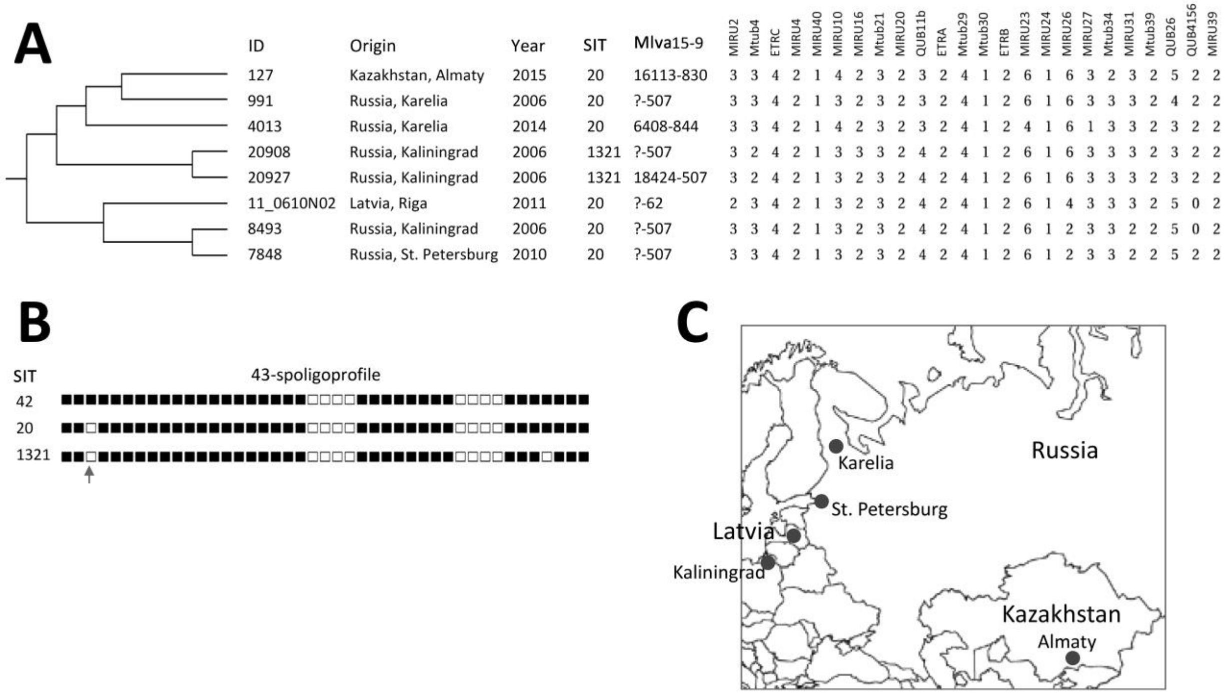

Appendix Figure 1. *Mycobacterium tuberculosis* RD-Rio strain in Kazakhstan. A) Section of the variable-number tandem-repeat–based dendrogram of the Latin-American-Mediterranean family of *Mycobacterium tuberculosis* RD-Rio strain with enlarged branch including SIT20 strain from Kazakhstan. All isolates were drug-susceptible. B) Binary spoligoprofiles of the studied strains. C) Locations of isolation of the studied strains.

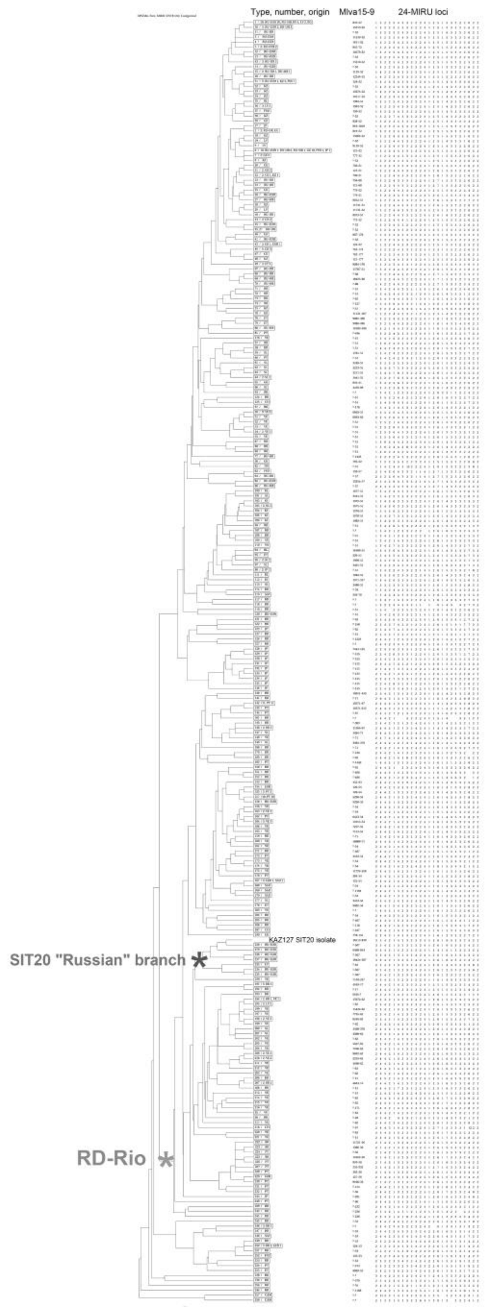

Appendix Figure 2. The UPGMA dendrogram of 259 VNTR types (357 LAM isolates) (Mokrousov et al., 2016) with SIT20 strain from Kazakhstan. Asterisks indicate RD-Rio sublineage and its branch with SIT20 strain from Kazakhstan (“SIT20 Russian branch”). The column “Type, number and origin” refers to the LAM types in Mokrousov et al. (2016). Mlva15–9 refers to nomenclature of MIRU-VNTRplus.org. Twenty-four VNTR loci are listed in the order in which they are found on chromosome, clockwise: 424, 577, 580, 802, 960, 1644, 1955, 2163b, 2165, 2401, 2996, 3192, 3690, 4052, 4156, 154, 2059, 2347, 2461, 2531, 2687, 3007, 3171, 4348.

Supplementary References

1. Allix-Béguec C, Harmsen D, Weniger T, Supply P, Niemann S. Evaluation and strategy for use of MIRU-VNTRplus, a multifunctional database for online analysis of genotyping data and phylogenetic identification of *Mycobacterium tuberculosis* complex isolates. J Clin Microbiol. 2008;46:2692–9. PubMed http://dx.doi.org/10.1128/JCM.00540-08

2. Demay C, Liens B, Burguière T, Hill V, Couvin D, Millet J, et al. SITVITWEB—a publicly available international multimarker database for studying *Mycobacterium tuberculosis* genetic diversity and molecular epidemiology. Infect Genet Evol. 2012;12:755–66. PubMed http://dx.doi.org/10.1016/j.meegid.2012.02.004

3. Gibson AL, Huard RC, Gey van Pittius NC, Lazzarini LC, Driscoll J, Kurepina N, et al. Application of sensitive and specific molecular methods to uncover global dissemination of the major RDRio sublineage of the Latin American–Mediterranean *Mycobacterium tuberculosis* spoligotype family. J Clin Microbiol. 2008;46:1259–67. PubMed http://dx.doi.org/10.1128/JCM.02231-07

4. Kamerbeek J, Schouls L, Kolk A, van Agterveld M, van Soolingen D, Kuijper S, et al. Simultaneous detection and strain differentiation of *Mycobacterium tuberculosis* for diagnosis and epidemiology. J Clin Microbiol. 1997;35:907–14. PubMed

5. Lazzarini LC, Huard RC, Boechat NL, Gomes HM, Oelemann MC, Kurepina N, et al. Discovery of a novel *Mycobacterium tuberculosis* lineage that is a major cause of tuberculosis in Rio de Janeiro, Brazil. J Clin Microbiol. 2007;45:3891–902. PubMed http://dx.doi.org/10.1128/JCM.01394-07

6. Mokrousov I, Rastogi N. Spacer-based macroarrays for CRISPR genotyping. Methods Mol Biol. 2015;1311:111–31. PubMed http://dx.doi.org/10.1007/978-1-4939-2687-9_7

7. Mokrousov I, Vyazovaya A, Narvskaya O. *Mycobacterium tuberculosis* Latin American–Mediterranean family and its sublineages in the light of robust evolutionary markers. J Bacteriol. 2014;196:1833–41. PubMed http://dx.doi.org/10.1128/JB.01485-13

8. Mokrousov I, Vyazovaya A, Iwamoto T, Skiba Y, Pole I, Zhdanova S, et al. Latin American–Mediterranean lineage of *Mycobacterium tuberculosis*: human traces across pathogen's phylogeography. Mol Phylogenet Evol. 2016;99:133–43. PubMed http://dx.doi.org/10.1016/j.ympev.2016.03.020

9. Rüsch-Gerdes S, Pfyffer GE, Casal M, Chadwick M, Siddiqi S. Multicenter laboratory validation of the BACTEC MGIT 960 technique for testing susceptibilities of *Mycobacterium tuberculosis* to

classical second-line drugs and newer antimicrobials. J Clin Microbiol. 2006;44:688–92. PubMed http://dx.doi.org/10.1128/JCM.44.3.688-692.2006

10. Supply P, Allix C, Lesjean S, Cardoso-Oelemann M, Rüsch-Gerdes S, Willery E, et al. Proposal for standardization of optimized mycobacterial interspersed repetitive unit-variable-number tandem repeat typing of *Mycobacterium tuberculosis.* J Clin Microbiol. 2006;44:4498–510. PubMed http://dx.doi.org/10.1128/JCM.01392-06

11. World Health Organization. Policy guidance on drug-susceptibility testing (DST) of second-line antituberculosis drugs. WHO/HTM/TB/2008.392. Geneva: The Organization; 2008.

<jrn>12. Zimenkov DV, Kulagina EV, Antonova OV, Zhuravlev VY, Gryadunov DA. Simultaneous drug resistance detection and genotyping of *Mycobacterium tuberculosis* using a low-density hydrogel microarray. J Antimicrob Chemother. 2016;71:1520–31. PubMed http://dx.doi.org/10.1093/jac/dkw015</jrn>
